# Exploring the Factors Contributing to the High Ultimate pH of Broiler Pectoralis Major Muscles Affected by Wooden Breast Condition

**DOI:** 10.3389/fphys.2020.00343

**Published:** 2020-05-08

**Authors:** Giulia Baldi, Con-Ning Yen, Morgan R. Daughtry, Jocelyn Bodmer, Brian C. Bowker, Hong Zhuang, Massimiliano Petracci, David E. Gerrard

**Affiliations:** ^1^Department of Agricultural and Food Sciences, University of Bologna, Bologna, Italy; ^2^Department of Animal and Poultry Sciences, Virginia Polytechnic Institute and State University, Blacksburg, VA, United States; ^3^US National Poultry Research Center, Quality & Safety Assessment Research Unit, Athens, GA, United States

**Keywords:** wooden breast, post-mortem metabolism, pH, sarcomere length, glycolysis

## Abstract

The elevated ultimate pH (pH_*u*_) found in wooden breast (WB) meat suggests an altered muscular energetic status in WB but also could be related to a prematurely terminated post-mortem pH decline. The aims of this study were to explore the factors contributing to the elevated pH_*u*_ and establish whether the occurrence of WB defect alters muscle post-mortem carbohydrate metabolism and determine if the contractile apparatus reflects such changes. A total of 24 carcasses from Ross 308 male chickens were obtained from a commercial producer and harvested using commercial processing procedures. Carcasses were categorized into unaffected (NORM) and WB groups (*n* = 12 each), and samples were collected from cranial bone-in pectoralis major (PM) muscles at 15 min and 24 h post-mortem for the determination of pH, glycolytic metabolites, adenonucleotides, buffering capacity, phosphofructokinase (PFK) activity, and *in vitro* pH decline. Twenty-four additional deboned PM samples (12 NORM and 12 WB) were collected from the same processing plant to assess muscle histology and sarcomere length at four different locations throughout the PM muscle. Data show that the reduced glycolytic potential of WB muscles only partially explains the higher (*P* < 0.001) pH_*u*_ of WB meat, as residual glycogen along with unaltered PFK activity suggests that neither glycogen nor a deficiency of PFK is responsible for arresting glycolysis prematurely. The dramatic reduction in ATP concentrations in the early post-mortem period suggests a defective ATP-generating pathway that might be responsible for the reduced pH decline in WB samples. Further, the addition of excess of ATPase extended post-mortem glycolysis of WB meat in an *in vitro* glycolytic system. WB-affected samples have longer (*P* < 0.001) sarcomeres compared to NORM, indicating the existence of compromised energy-generating pathways in myopathic muscles that may have had consequences on the muscle contraction and tension development, as *in vivo*, also during the post-mortem period. Considering the overall reduced glycolytic potential and the myodegenerative processes associated with WB condition, we speculate that the higher pH_*u*_ of WB meat might be the outcome of a drastically impaired energy-generating pathway combined with a deficiency and/or a dysfunction of muscle ATPases, having consequences also on muscle fiber contraction degree.

## Introduction

In an attempt to address the ever-growing demand for poultry meat, selection pressures on chicken genetics have resulted in huge gains in animal growth rates, feed conversion, and enhanced growth of the pectoralis major (PM) muscle chickens, which represents the most profitable portion in the broiler industry, at least in Western countries (Petracci et al., 2019). As a result, however, the incidence of metabolic and growth-related disorders affecting PM muscles of broiler chickens has increased, suggesting genetic improvements have given rise to the occurrence of muscular abnormalities (Griffin et al., 2018; Velleman, 2019). Among these, wooden breast (WB) has been reported in several plants all over the world with an occurrence up to 50% of affected individuals within the same flock (Griffin et al., 2018; Livingston et al., 2018), resulting in costly economic deficits (Kuttappan et al., 2017b). This global widespread muscular abnormality appears as either a focally or diffusely palpably firm consistency in the breast muscle, which is undesirable, pale, out-bulging, and often displays superficial exudate and petechiae (Sihvo et al., 2014). Microscopically, WB-affected muscles exhibit an altered muscular architecture, showing a diffuse thickening of the endomysial and perimysial connective tissue associated with several degrees of fiber necrosis, inflammatory cell infiltrations, as well as extreme collagen and fat deposition (Sihvo et al., 2014; Soglia et al., 2016; Clark and Velleman, 2017). Moreover, selection for enhancing breast muscle mass has increased muscle fiber size through greater post-hatch hypertrophic growth (Clark and Velleman, 2017; Velleman, 2019), thus reducing capillary-to-fiber ratio and resulting in severe circulatory insufficiency (i.e., hypoxia), oxidative stress mechanisms, as well as remarkable alterations in muscle metabolism (Sihvo et al., 2018). Indeed, metabolomic studies provide evidence of dysregulated lipid and carbohydrate metabolism in affected birds (Mutryn et al., 2015; Abasht et al., 2016; Papah et al., 2017) to such a degree that the elevated ultimate pH (pH_*u*_) of WB meat is usually considered a hallmark of this muscular abnormality. However, even though many aspects of WB abnormality have been investigated at the molecular level, to the best of our knowledge, there has not been any indication concerning post-mortem metabolism of WB-affected muscles. Considering that glycogen is not usually a limiting factor in chicken PM muscle (Chauhan and England, 2018), the high pH_*u*_ of WB meat may be related to a prematurely terminated post-mortem pH decline. Thus, having information about the enzymes and metabolites involved in post-mortem glycolysis might be crucial to understand the reasons behind the altered energetic status of myopathic muscles and provide new insights into this condition. Moreover, since the rate and the extent of post-mortem muscular acidification influence the degree of myofibrillar contraction (Ertbjerg and Puolanne, 2017), it could be speculated that the extreme stiffness of WB muscles might be partially due to a hypercontraction of sarcomeres caused by an abnormal acidification process or premature rigor formation. Within this context, the present study aimed at widening the knowledge about WB condition by exploring the factors contributing to the elevated pH_*u*_ and establishing whether its occurrence exerts an effect on muscle post-mortem glycolysis and muscle fiber contraction.

## Materials and Methods

### Muscle Sampling

**FIGURE 1:**
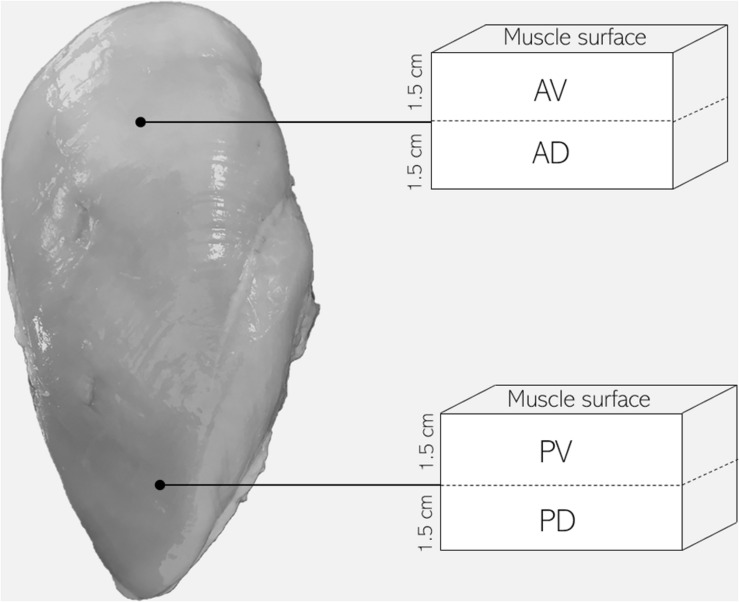
Schematic diagram of pectoralis major muscle sampling location for the evaluation of muscle histology and sarcomere length (AV, anteroventral; AD, anterodorsal; PV, posteroventral; PD, posterodorsal).

A total of 24 carcasses were obtained from the same flock of broiler chickens (Ross 308 strain, males, 48 days of age, 3.0–3.5 kg live weight), reared and harvested using standard US commercial procedures. Carcasses were selected immediately after evisceration (∼15 min post-mortem) from the line of a commercial broiler processing plant and categorized by experienced personnel in unaffected (NORM) and WB (*n* = 12/group) based on the criteria proposed by Sihvo et al. (2014). More specifically, WB carcasses were selected based on palpable hardness and muscle rigidity throughout the whole PM muscle, picking the most severe cases. Carcasses were stored in cold storage room at 4°C, and samples of about 1 cm^3^ were collected from the cranial position of bone-in PM muscles of each carcass at both 15 and 1,440 min (24 h) post-mortem, snap frozen in liquid nitrogen, and stored at −80°C until analyses. Samples were then used for the assessment of pH, glycolytic metabolites, adenine nucleotides, buffering capacity, phosphofructokinase (PFK) activity, and *in vitro* pH decline. Further, 24 additional deboned PM muscles were collected from the deboning line of the same commercial broiler processing plant at 3 h post-mortem and categorized into NORM and WB (*n* = 12/group) following the same criteria described before. Fillets were stored at 4°C until 24 h post-mortem (i.e., until rigor mortis resolution), and samples from four different locations throughout the PM muscle were obtained in order to evaluate the spatial effects of WB condition on muscle histology and sarcomere length. Specifically, samples were collected from anteroventral, anterodorsal, posteroventral, and posterodorsal regions of WB-affected PM muscles, while samples from NORM breast muscles were acquired from anteroventral section (Figure 1). Samples belonging to anterodorsal and posterodorsal positions were collected by making an incision in the breast muscle in order to obtain samples located 1.5 cm underneath the anteroventral and posteroventral samples, respectively. All the aspects of rearing, handling, transportation, and harvest of birds were accomplished under US laws.

### pH and Glycolytic Metabolites

Samples were processed as described by Matarneh et al. (2018) with slight modifications. Briefly, frozen 15- and 1,440-min PM muscle samples (*n* = 12/group) were powdered under liquid nitrogen using a mortar and pestle, and three aliquots of approximately 0.1 g were collected. Powdered samples were lysed using a Tissue Lyser II system (Qiagen, Boston, MA, United States) in 0.8 ml of ice-cold 5 mM sodium iodoacetate and 150 mM KCl solution (pH = 7.0). Following centrifugation at 17,000 × *g* for 5 min and equilibration to 25°C, pH of supernatants was directly measured using an Orion Ross Ultra pH glass electrode (Thermo Scientific, Pittsburgh, PA, United States). Samples designated for glucose, glucose-6-phosphate (G6P), lactate, and adenine nucleotides (ATP, ADP, AMP, and IMP) were lysed in 1 ml of ice-cold 0.5M perchloric acid and incubated on ice for 20 min. Homogenates were centrifuged at 17,000 × *g* for 5 min, then supernatants were transferred into new tubes and neutralized with 2M KOH. As for muscle glycogen determination, another sample was lysed in 1 ml of 1.25M HCl, heated at 90°C for 2 h, and centrifuged at 17,000 × *g* for 5 min. Following, supernatants were transferred into new tubes and neutralized with 1.25M KOH. Glycogen, glucose, G6P, and lactate were determined using enzymatic methods modified for a 96-well plate as described by Hammelman et al. (2003), and glycolytic potential (GP) was calculated as suggested by Scheffler et al. (2013) following the equation: GP (μmol/g) = 2 ^∗^ (glucose + G6P + glycogen) + lactate. Adenine nucleotide concentrations were quantified using HP Agilent 1100 HPLC system (Agilent Technologies, Santa Clara, CA) and external standards.

### *In vitro* Glycolysis Model

Frozen 15-min NORM and WB samples (n = 6/group) were powdered in liquid nitrogen and homogenized at 1:10 (wt/vol) in a glycolysis buffer containing 5 mM MgCl_2_, 10 mM Na_2_HPO_4_, 60 mM KCl, 5 mM Na-ATP, 0.5 mM ADP, 0.5 mM NAD^+^, 25 mM carnosine, 30 mM creatine, 40 mM glycogen, and 10 mM sodium acetate (pH 7.4) (England et al., 2014). In order to test the effect of ATPase on muscle acidification, either 0 or 2 U/ml of ATPase were incorporated into the *in vitro* model. Reaction vessels were incubated at 25°C for the duration of the trial. Aliquots for pH determination were removed from reaction vessels at 0, 15, 30, 120, 240, and 1,440 min and homogenized at 1:4 (vol/vol) ratio with a 25 mM sodium iodoacetate and 750 mM KCl solution (pH = 7.0). Samples were centrifuged at 17,000 × *g* for 5 min, equilibrated to 25°C, and pH was measured directly using an Orion Ross Ultra pH glass electrode (Thermo Scientific, Pittsburgh, PA, United States).

### Phosphofructokinase Activity Assay

PFK activity of both NORM and WB PM muscles (*n* = 6/group) was determined according to the procedures described by England et al. (2014). Briefly, ∼0.1 g of the 15-min samples was homogenized at 1:10 (wt/vol) in ice-cold 100 mM K_2_HPO_4_ solution (pH = 7.4). Subsequently, aliquots of tissue homogenate were added to a reaction buffer containing 120 mM MES, 3.2 mM MgSO_4_, 2 mM ATP, 1 mM NADH, 3 mM fructose-6-phosphate, 2 U/ml triosephosphate isomerase, 1 U/ml glycerol-3-phosphate dehydrogenase, and 1 U/ml aldolase (pH = 7.0). Enzymatic activity was measured spectrophotometrically at 340 nm and reported as nmol NADH × min^–1^ × g^–1^.

### Buffering Capacity

Buffering capacity of NORM and WB samples (*n* = 12/group) was determined according to Matarneh et al. (2015). Briefly, ∼1 g of the 1,440 min meat was homogenized in 5 mM sodium iodoacetate and 150 mM KCl solution (pH = 7.0) at 1:10 ratio (wt/vol). After equilibration to 25°C, samples were titrated using 0.1M NaOH. Samples pH was measured using an Orion Ross Ultra pH glass electrode (Thermo Scientific, Pittsburgh, PA, United States) and buffering capacity was calculated as follows: buffering capacity = ΔB/ΔpH, where ΔB is the increment of base expressed as μmol NaOH/g of tissue, and ΔpH is the corresponding pH variation.

### Histology

Muscle samples of approximately 1 cm^3^ were removed from anteroventral, anterodorsal, posteroventral, and posterodorsal regions of WB de-boned chicken PM muscles (*n* = 12) at 24 h post-mortem. Meat samples were placed in 10% (vol/vol) buffered formalin fixative (pH = 7) and stored at 4°C. Subsequently, samples were dehydrated in a graded series of ethanol, oriented for cross-sectional fiber sectioning, and paraffin-embedded. Paraffin blocks were cut at 6 μm with a microtome, mounted on saline-coated microscope slides, and hematoxylin and eosin stained. In more detail, muscle sections were stained with hematoxylin for 6 min, rinsed with running deionized water, submerged in eosin for 2 s, and rinsed again with deionized water. Slides were then rinsed in 50 and 70% ethanol 10 times, then in 95% ethanol for 30 s, then in 100% ethanol for 60 s. Muscle sections were then rinsed in xylene seven times, dried with a Kimwipe, and mounted. Digital photomicrographs were taken using a Nikon ECLIPSE microscope (Nikon Instruments, Inc., United States) equipped with a 40 × objective.

### Sarcomere Length

Muscle samples of approximately 1 cm^3^ and oriented along the muscle fibers were collected at 24 h post-mortem from the anteroventral zone of NORM PM (*n* = 6) and anteroventral, anterodorsal, posteroventral, and posterodorsal locations of WB PM de-boned muscles (*n* = 6/location) used for histology evaluation, placed in 10% (vol/vol) buffered formalin fixative (pH = 7.0) and stored at 4°C. Subsequently, samples were dehydrated in a graded series of ethanol, oriented for longitudinal fiber sectioning, and paraffin-embedded. Paraffin blocks were cut at 3 μm with a microtome, mounted on saline-coated microscope slides, and hematoxylin and eosin stained. Each slide contained a minimum of two sections. Digital photomicrographs were taken with Nikon ECLIPSE microscope (Nikon Instruments, Inc., United States) equipped with an oil immersion 100 × objective and processed using ImageJ software NIH Image. For each section, 15 myofibrils at least 5 sarcomeres long were selected for the assessment of sarcomere length, which was evaluated as the ratio between the total length of the myofibril (i.e., the distance between A bands) and the number of sarcomeres.

### Statistical Analysis

Overall data from the experiment were analyzed using the one-way ANOVA option of the GLM procedure of SAS software (SAS Institute, Inc., United States). Data concerning pH, glycolytic metabolites, and adenine nucleotides were analyzed using the occurrence of WB defect as the main effect within each sampling time (15 and 1,440 min). As for the *in vitro* study, the statistical model included the addition of ATPase to the meat as the main effect for each sampling time (0, 15, 30, 120, 240, and 1,440 min), while results concerning buffering capacity between NORM and WB groups were compared within each pH value. Data regarding sarcomere length detected from AV sampling position were analyzed by comparing unaffected and affected samples, while the spatial influence of WB through affected PM muscles was evaluated using sampling location (anteroventral, anterodorsal, posteroventral, and posterodorsal) as the main effect. Data regarding the effect of WB condition on muscle glycolytic potential and PFK activity were analyzed by comparing NORM and WB experimental groups. Means were then evaluated using Tukey’s multiple range test of the GLM procedure and considered significant at *P* < 0.05. All data are expressed as means ± SEM.

## Results and Discussion

### pH and Glycolytic Metabolites

**FIGURE 2:**
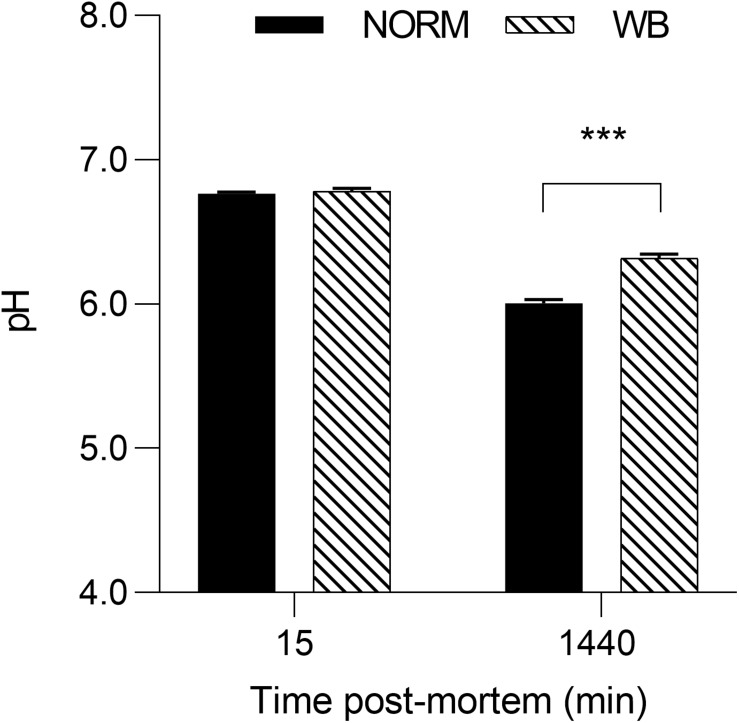
Average pH of unaffected (NORM) and wooden breast (WB) broiler pectoralis major muscles (*n* = 12/group) at 15 and 1,440 min post-mortem. Data represent means ± SEM. Asterisks indicate significant difference within the time point (***P < 0.001).

**FIGURE 3:**
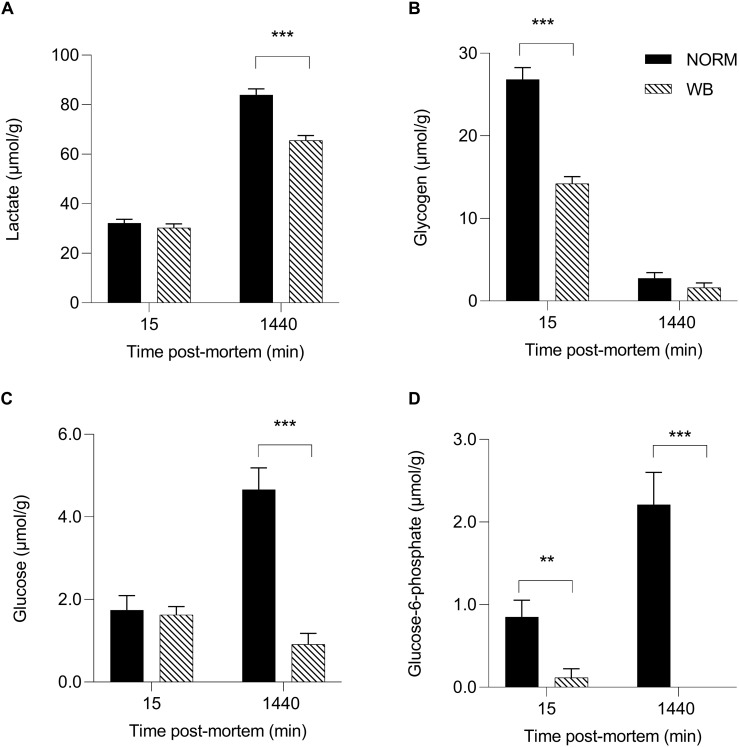
Average lactate (**A**, μmol/g), glycogen (**B**, μmol/g), glucose (**C**, μmol/g), and glucose-6-phosphate (**D**, μmol/g) of unaffected (NORM) and wooden breast (WB) broiler pectoralis major muscles (*n* = 12/group) at 15 and 1,440 min post-mortem. Data represent means ± SEM. Asterisks indicate a significant difference within the time point (***P < 0.001; **P < 0.01).

Given that selection programs have exerted significant consequences on muscle metabolism (Petracci et al., 2017), understanding post-mortem glycolysis in WB-affected birds may be useful in providing greater understanding into this muscular abnormality. Results concerning the effect of WB condition on muscle pH and glycolytic metabolites are displayed in Figures 2, 3, respectively. While no differences were detected at 15 min post-mortem, a significantly higher pH_*u*_ of 6.32 (*P* < 0.001) was observed in muscles affected by WB. The higher pH_*u*_ of WB meat found within this experiment and confirmed by previous studies (Mudalal et al., 2015; Kuttappan et al., 2017b; Baldi et al., 2019; Tasoniero et al., 2019) is commonly considered a hallmark of this myopathy, whereas the regular pH_*u*_ values of chicken pectoral muscles range typically from 5.8 to 5.9 (Petracci et al., 2017).

**FIGURE 4:**
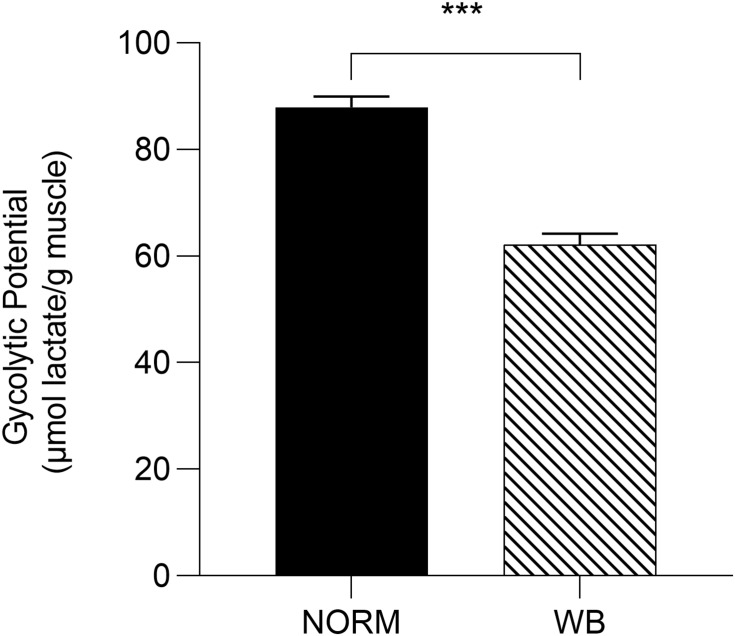
Glycolytic potential (μmol lactate/g meat) of unaffected (NORM) and wooden breast (WB) broiler pectoralis major muscles (*n* = 12/group). Data represent means ± SEM. Asterisks indicate a significant difference between experimental groups (***P < 0.001).

Accordingly, lactate contents followed pH decline, with WB muscles accumulating less lactate by 1,440 min post-mortem (*P* < 0.001; Figure 3A). Reduced lactate formation was also found in severely affected WB chickens by Malila et al. (2019) and might be explained with the loss of lactate dehydrogenase (LDH) enzyme from abnormal muscle fibers (Abasht et al., 2016). Moreover, it has been recently suggested that decreased lactate concentrations found in WB meat might be linked to an alteration of genes involved in lactate metabolism due to chronic hypoxic conditions responsible for exportation of lactate out of muscle cells (Zhao et al., 2019). However, lower lactate levels at 1,440 min along with the higher pH_*u*_ of WB samples suggest premature cessation of post-mortem metabolism, which is often thought to be associated with the depletion of muscular glycogen and/or an indication of a reduced amount prior to harvest. Curiously, glycogen content was found to be lower in WB-affected muscles at 15 min post-mortem (*P* < 0.001; Figure 3B), corroborating the findings of Abasht et al. (2019) and Malila et al. (2019), but no differences were detected at 1,440 min. It has been reported that glycogen storage in breast muscle decreases with the increase in muscle fiber size (Berri et al., 2007), suggesting that selection for hybrids with high growth rate and muscle yield (mainly achieved through fiber hypertrophy) might lead to reduced muscular glycogen concentrations in modern broiler breast muscles. On the other hand, G6P concentrations at both 15 and 1,440 min were likewise reduced in WB meat (*P* < 0.01 and *P* < 0.001, respectively; Figure 3D), while glucose concentration was found to be higher (*P* < 0.001; Figure 3C) in NORM samples at 1,440 min. As a direct consequence of the diminished content of glycolytic metabolites, WB-affected muscles exhibit lower glycolytic potentials (*P* < 0.001), reduced by 30% when compared to NORM samples (Figure 4). Lower glycolytic potential in WB muscles indicates reduced substrate flux through glycolysis and, therefore, lower production of both H^+^ and lactate, causing a higher pH_*u*_ of the forthcoming meat. These results confirmed what has been extensively reported in previous genetic and proteomic studies (Abasht et al., 2016; Zambonelli et al., 2016; Kuttappan et al., 2017a; Malila et al., 2019), suggesting that muscles showing severe myopathic lesions are usually characterized by downregulated carbohydrate metabolism and, as a consequence, a reduced content of glycolytic metabolites. Indeed, overall data collected within this study corroborate altered glycogen and glucose metabolic pathways. Considering the extreme hypoxic conditions and inflammatory processes taking place in WB muscles, the reduced content of glycolytic metabolites could be explained by a rerouting of the carbohydrate flow from glycolysis to other metabolic pathways in order to contrast muscle inflammation (Lake et al., 2019). Finally, the presence of residual glycogen in WB muscle found at 24 h post-mortem suggests that glycolysis did not arrest prematurely due to substrate deficiency.

### Phosphofructokinase Activity

**FIGURE 5:**
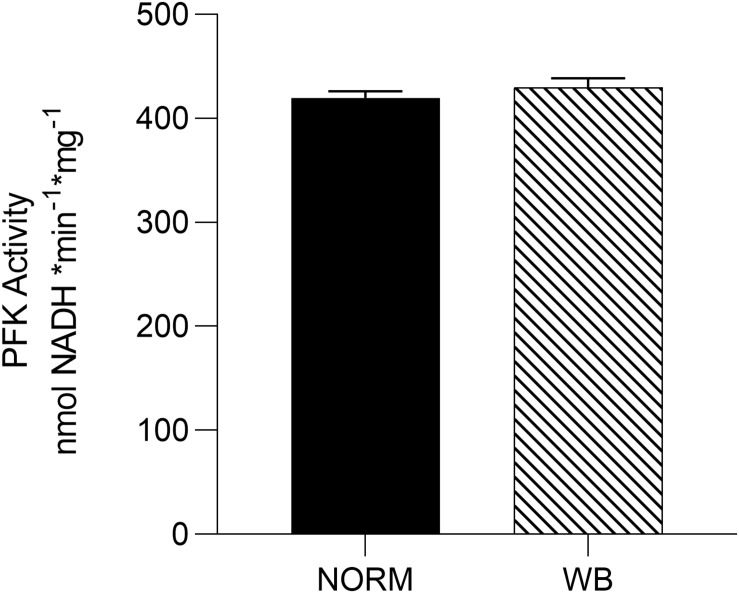
Average phosphofructokinase (PFK) activity [nmol nicotinamide adenine dinucleotide (NADH) * min^– 1^ * g^–1^] of unaffected (NORM) and wooden breast (WB) pectoralis major muscles (*n* = 6/group). Data represent means ± SEM.

In the presence of residual glycogen, Matarneh et al. (2018) reported that pH_*u*_ is determined by the activity of PFK, a key regulatory enzyme in the glycolytic pathway that irreversibly catalyzes the conversion of fructose 6-phosphate into fructose 1,6-biphosphate. Results concerning PFK activity are reported in Figure 5. Surprisingly, no differences were detected in PFK activity between affected and unaffected muscles, meaning that PFK is not responsible for arresting post-mortem glycolysis in WB-affected samples. However, Minchenko et al. (2002) reported that in hypoxic conditions, PFK plays a key role as a regulator of glucose consumption in order to maintain energetic homeostasis. With this in mind, the unaltered PFK activity found in WB muscles might represent an attempt of muscle cells to combat hypoxia by regulating glucose metabolism through different mechanisms. Further explanation for this outcome remains to be studied.

### Adenine Nucleotides

**FIGURE 6:**
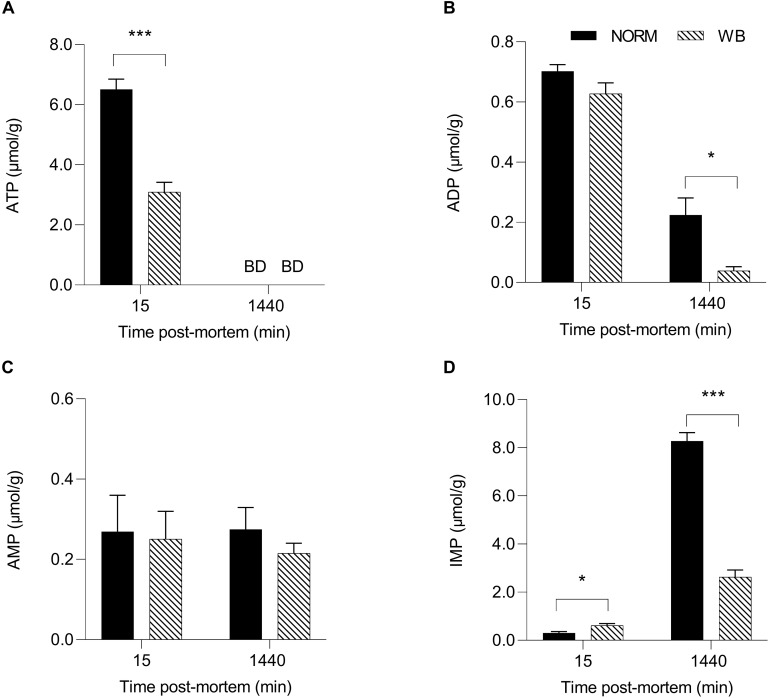
Average ATP (**A**, μmol/g), ADP (**B**, μmol/g), AMP (**C**, μmol/g), and IMP (**D**, μmol/g) of unaffected (NORM) and wooden breast (WB) broiler pectoralis major muscles (*n* = 12/group) at 15 and 1,440 min post-mortem. Data represent means ± SEM. Asterisks indicate a significant difference within the time point (***P < 0.001; *P < 0.05). BD, below limit of detection.

In an attempt to better understand post-mortem metabolism in WB-affected muscles, adenine nucleotides levels were also measured. England et al. (2014) reported that in some cases, the depletion of adenosine nucleotides (ATP, ADP, IMP, and AMP) could arrest glycolysis while PFK is still functioning. Results concerning adenine nucleotides levels are shown in Figure 6. ATP content (Figure 6A) was 50% lower in WB-affected muscle at 15 min post-mortem (*P* < 0.001) compared to NORM (3.10 vs. 6.51 μmol/g). Intriguingly, WB-affected samples had higher IMP levels at 15 min post-mortem. No differences were detected in AMP concentrations (Figure 6C), while both ADP (Figure 6B) and IMP (Figure 6D) contents were lower in WB muscles at 1,440 min post-mortem (*P* < 0.05 and < 0.001, respectively). The lower IMP content found at 1,440 min within this study corroborates findings from Abasht et al. (2016) and Soglia et al. (2019), who suggest that lower levels of adenosine nucleotides along with higher levels of catabolites such as xanthine and urate might explain a greater nucleotide degradation in affected muscles. On the other hand, lower ADP concentration of WB muscle at 1,440 min post-mortem might be the result of WB muscles attempting to combat the deficit in ATP concentrations by generating ATP from two molecules of ADP. This reaction also produces one molecule of AMP, which in turn could be deaminated to IMP and NH_3_. This might also explain the higher content of IMP in WB muscles at 15 min post-mortem, as muscle attempts to compensate for the lack of ATP in the first minutes after the death. However, the reasons behind the drastic reduction in ATP content might be different. The first hypothesis is related to creatine, a compound that plays a key role in muscular energy metabolism since it is directly involved in ATP synthesis *in vivo* (Mora et al., 2008). A study conducted by Sundekilde et al. (2017) revealed that dystrophic muscles present significantly lower creatine content, thus suggesting a perturbation in the energy-generating pathway. This result has also been confirmed by Soglia et al. (2019) and Wang et al. (2019) who found remarkably reduced creatine content in muscles affected by WB. Thus, being creatine present in lower concentrations in myopathic muscles, it is reasonable to speculate that the ATP-generating pathway might be compromised, resulting in reduced ATP concentrations in the early post-mortem. The second hypothesis concerns the impaired mitochondrial functionality of WB muscles found in a recent study (Sihvo et al., 2018), where the authors reported that WB-affected samples exhibit mitochondrial swelling, vacuolation, and cristae loss, clear indicators of osmotic imbalance and hypoxia. The same authors also suggest that muscles that experienced distress, such as hypoxia at any time during the lifetime of an animal, might be more prone to develop post-mortem alterations. Under these circumstances, since *in vivo* ATP synthesis happens through mitochondrial respiration, it is reasonable to postulate that the reduced ATP content found within this study might be partially due to the impaired mitochondria functionality found in WB-affected muscles. Aside from these theories, it is noteworthy to highlight that the severe histopathological lesions associated with WB condition (which will be discussed later in the paper) might represent the most logical explanation for the reduced nucleotide content found within affected samples, since the presence of necrotic fibers may have resulted in a lower ATP content. However, the drastic reduction in ATP concentrations in the early post-mortem period, along with the remarkably lower glycolytic potential of myopathic muscles, undoubtedly provoked a reduced extent of pH decline in WB samples (i.e., higher pH_*u*_). ADP and AMP were not depleted by 1,440 min post-mortem, suggesting that adenine nucleotides alone were not likely responsible for arresting post-mortem glycolysis.

### Buffering Capacity

**FIGURE 7:**
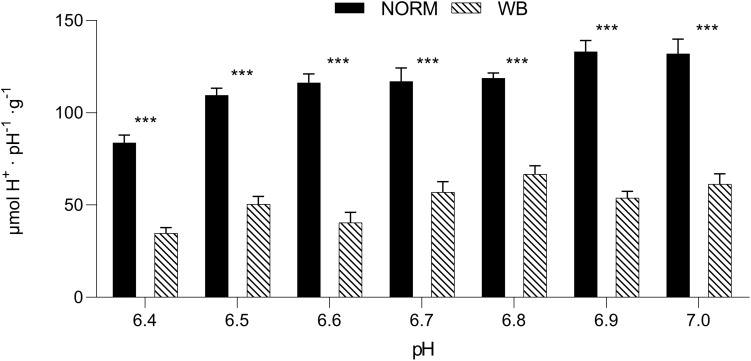
Buffering capacity (μmol H^+^⋅pH^–1^⋅g^–1^) (pH range 6.4–7.0) in unaffected (NORM) and wooden breast (WB) pectoralis major muscles (*n* = 12/group). Data represent means ± SEM. Asterisks indicate a significant difference within the same pH value (***P < 0.001).

The pH_*u*_ of a muscle also depends on the buffering systems of the muscle itself (Laack et al., 2001), thus, exploring muscle’s buffering capacity may provide further knowledge about WB post-mortem glycolysis. Buffering capacity was significantly affected by the occurrence of WB defect (Figure 7). WB samples exhibited significantly lower buffering capacity (*P* < 0.001) for each pH value considered within the range of 6.4–7.0, meaning that WB-affected muscles have a lower ability to buffer H^+^ produced during post-mortem glycolysis. However, a reduced buffering capacity should have implied the achievement of a lower pH_*u*_, yet in WB meat, this condition is coupled with an overall reduced glycolytic potential that consequently led to decreased H^+^ accumulation. Nevertheless, muscle’s ability to buffer the acidic end-products of glycolysis is due by half to myofibrillar proteins, while phosphate compounds and histidine-containing dipeptides (e.g., anserine and carnosine) contributed to the other half (Matarneh et al., 2017). In more detail, anserine and carnosine, in light of their pKa (6.38 and 7.04, respectively), are considered the most important compounds for the maintenance of muscle homeostasis and prevention of tissue damage (Davey, 1960; Jung et al., 2013). As *in vivo*, also during the conversion of muscle to meat histidine dipeptides play a determinant role in counteracting rapid changes in pH. However, a recent study conducted by Soglia et al. (2019) revealed that the concentrations of these compounds were significantly lower in WB muscles compared to unaffected ones. More specifically, anserine and carnosine levels were reduced by 37.2 and 46.1%, respectively, in myopathic muscles, corroborating data from Sundekilde et al. (2017). Within this context, it is reasonable to assume that the reduced content of buffering compounds coupled with compromised muscle fibers structure and functionality (Sihvo et al., 2014; Velleman and Clark, 2015) severely affected the buffering capacity of muscles affected by WB condition.

### *In vitro* pH Decline

**FIGURE 8:**
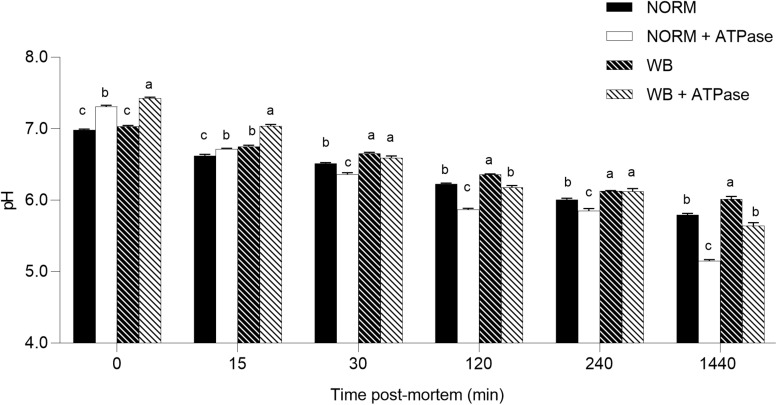
Average pH of the *in vitro* model (*n* = 6/group). Data represent means ± SEM. (a–c) Means lacking a common letter significantly differ within a time point (*P* < 0.05).

Considering the overall dataset, the reduced content of glycolytic metabolites only partially explains the higher pH_*u*_ detected in WB muscle. Residual glycogen found in WB samples at 1,440 min post-mortem along with unaltered PFK activity suggests neither glycogen content nor PFK activity was responsible for arresting glycolysis. On the other hand, the absence of G6P at 1,440 min raises the possibility that glycogenolysis was somehow inhibited. Thus, since post-mortem glycolysis is regulated by ATPase activity (Scopes, 1974), an *in vitro* system was used to simulate muscle acidification with or without excess ATPase in order to further test the factors contributing to the higher pH_*u*_ in WB meat. Using this system, we were able to compare pH decline in affected and unaffected samples with or without 2 U ATPase under the same environment (Figure 8). Because muscle samples from both groups were homogenized in the same buffer, differences in pH decline should be a function of the incorporated muscle tissue. The pH of the *in vitro* system was significantly affected by the addition of ATPase in the buffer. At both 0 and 15 min post-mortem, the addition of ATPase significantly raised muscular pH. NORM + ATPase and WB + ATPase groups showed significantly higher pH values when compared to their counterparts without ATPase. Scopes (1974) explained that the increased muscle pH with the addition of ATPase is due to immediate use of H^+^ ions with ADP and phosphocreatine to produce creatine and ATP prior to anaerobic glycolysis. Thus, for every re-phosphorylation of ADP by phosphocreatine, a H^+^ ion is consumed by the creatine kinase reaction, causing a slight increase of the pH (Scopes, 1974). However, after an initial slower rate, addition of ATPase increased the rate of pH decline of NORM + ATPase samples, which exhibited the lowest pH values for each time point. This is likely due to the addition of an excess ATPase to the system that stimulated additional glycolytic flux and consequently results in a lower pH. After 30 min post-mortem, the addition of ATPase appeared to increase the rate of pH decline in WB muscle and, at 1,440 min, reaction vessels containing WB muscle with an excess of ATPase showed significantly lower values when compared to their counterparts without ATPase (5.67 vs. 6.01; *P* < 0.05). No statistically significant differences were detected between NORM and WB + ATPase groups at 1,440 min, suggesting that an excess of ATPase can extend *in vitro* post-mortem glycolysis in WB muscles. Considering the presence of significant damaged/abnormal myofibers in WB tissue, it is reasonable to hypothesize that a deficiency and/or a dysfunction of muscular ATPases might be one of the factors responsible for arresting post-mortem glycolysis prematurely in WB muscles. Further, given the abundance of myosin ATPase in skeletal muscle, ATP hydrolysis by myofibrillar component likely drives post-mortem metabolism (Ferguson and Gerrard, 2014). Thus, further studies should be conducted in order to investigate if post-mortem metabolism is repressed because of a deficiency rather than a reduced functionality of myosin ATPases.

### Histology

**FIGURE 9:**
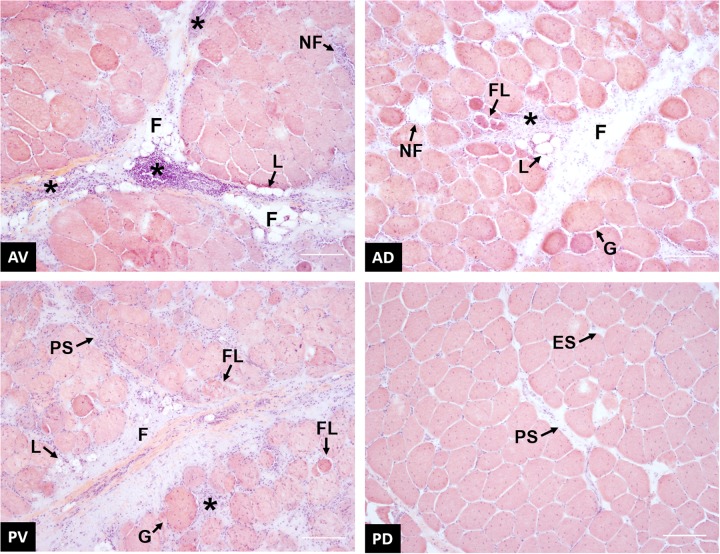
Representative images of wooden breast (WB)-affected samples collected from anteroventral (AV), anterodorsal (AD), posteroventral (PV), and posterodorsal (PD) positions of the pectoralis major muscle. * inflammatory cells infiltrate; F, proliferation of connective tissue; L, fat tissue deposition; NF, necrotic fiber; FL, fiber lysis; PS, perimysial spacing; ES, endomysial spacing; G, giant hypertrophic fiber. Scale bar = 150 μm.

Although the effect of WB abnormality on muscle fibers and microstructure has been deeply investigated, few studies take into consideration the evaluation of fiber morphology throughout different zones of PM muscle (Clark and Velleman, 2017; Soglia et al., 2017). Figure 9 contains representative images showing the morphological structure of anteroventral, anterodorsal, posteroventral, and posterodorsal regions of broiler PM affected by WB. In agreement with previous studies (Sihvo et al., 2014; Clark and Velleman, 2017; Soglia et al., 2017), anteroventral region exhibited severe myodegenerative lesions, including several extensive macrophage infiltrations, accumulation of fat (i.e., lipidosis) and connective tissue (i.e., fibrosis), as well as the presence of necrotic fibers surrounded by inflammatory cell infiltrates. The anteroventral region appears to have the highest degree of macrophage infiltration when compared to all other locations. Affected areas are demarcated by the replacement of damaged fibers with the deposition of connective tissue, causing a complete reorganization of the skeletal muscle structure. According to Sihvo et al. (2014), a severe thickening in the interstitial tissue separating muscle fiber bundles and nuclei internalization have been widely detected in anteroventral area. The main causes associated with this altered histological profile have been widely attributed to the reduced microcirculation due to excessive development of the pectoral muscle, which consequently leads to impaired muscle fiber metabolism and oxygen supply (Zambonelli et al., 2016; Sihvo et al., 2017). Among published studies, there are contrasting results about the effect of WB defect on the histologic characteristics of breast muscle’s inner cranial region probably due to the severity grade of the myopathy. According to Soglia et al. (2017), the anterodorsal zone exhibited several myodegenerative traits, such as fibrosis, lipidosis, necrotic fibers surrounded by macrophage infiltrations, and the presence of giant and hypercontracted fibers. Thickening of the endomysial spaces with deposition of connective tissue was observed as well. Moreover, the existence of fibers characterized by small diameters could be the result of fiber lysis, a process taking place in the muscle as a reparative and/or adaptive mechanism against fiber necrosis and degeneration. Additionally, Clark and Velleman (2017) found the presence of regenerating myofibers in the same region of PM muscles affected by WB. In the most severe cases, WB abnormality affects both the cranial and the caudal regions of superficial pectoral muscle (Kuttappan et al., 2016). From histological examinations conducted within this study, the posteroventral region showed an overall compromised muscular structure architecture, nearly comparable to what was observed for the anteroventral region. In addition to fibrosis, lipidosis, and fiber lysis, thickening of the perimysial and endomysial spaces and inflammatory cell infiltrations surrounding necrotic fibers have been observed. These findings are quite conflicting with what was observed in the same broiler PM region by Clark and Velleman (2017), who reported that the histological profile of the posteroventral area was just slightly affected by the occurrence of WB myopathy. However, the divergence detected between these studies might be ascribable to the different severity grades of WB condition and/or slaughter age of animals. Furthermore, based on the current knowledge, no studies have been conducted to investigate the histological traits of caudal deep section of PM muscle affected by WB. Contrary to other locations, the histological profile of posterodorsal region did not appear as severely compromised as other sampling locations. Neither infiltrating inflammatory cells nor fiber necrosis were found, while endomysial and perimysial spacing, as well as nuclei internalization and endomysial connective tissue proliferation, were evident. Generally, when compared to their superficial counterparts, the histological profile of the deep sections appeared to be less impacted by the occurrence of WB. The different degrees of myodegeneration between the upper and the inner sections of the pectoral muscle might be attributed to the different distance from the blood vessels responsible for oxygen translocation (Soglia et al., 2017). The greater physical proximity of the muscle fibers to capillaries and vessels might have resulted in a better oxygenation of the muscular tissue, thus limiting muscle damages related to oxidative stress.

### Sarcomere Length

**FIGURE 10:**
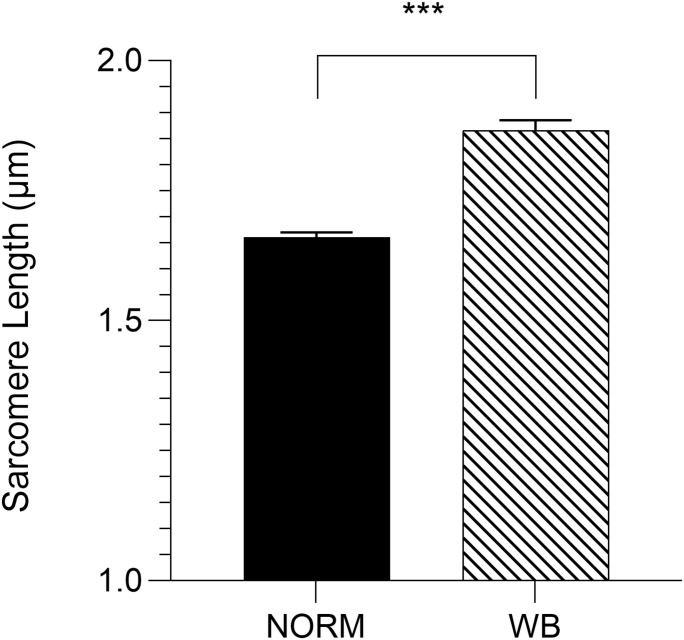
Average sarcomere length (μm) assessed in the anteroventral region of unaffected (NORM) and wooden breast (WB)-affected pectoralis major muscles (*n* = 6/group). Data represent means ± SEM. ***P < 0.001.

**FIGURE 11:**
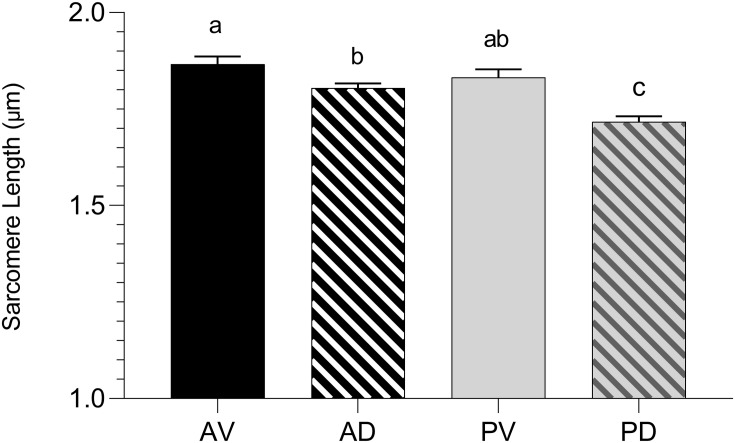
Average sarcomere length (μm) assessed in the anteroventral (AV), anterodorsal (AD), posteroventral (PV), and posterodorsal (PD) positions of pectoralis major muscles affected by WB myopathy (*n* = 6/position). Data represent means ± SEM. (a–c) Means lacking a common letter significantly differ (*P* < 0.05).

Because the rate and the extent of post-mortem muscular acidification influence the degree of myofibrillar contraction (Ertbjerg and Puolanne, 2017), it has been speculated if the extreme stiffness of WB muscles could be partially due to a hypercontraction of sarcomeres caused by an abnormal acidification process. Contrary to what was expected, WB samples belonging to the anteroventral region (superficial, cranial) showed significantly (*P* < 0.001) longer sarcomeres when compared to samples collected from the same area of NORM PM muscles (1.86 versus 1.66 μm, respectively; Figure 10). This result corroborates data from Tijare et al. (2016) and Sun et al. (2018), where investigators suggested that the longer sarcomeres detected in the cranial region of WB muscle might be due to the increased collagen content and the loss of muscle fiber vitality that might prevent shortening. In light of the results collected within this study, it could be postulated that the overall reduced content of ATP detected among WB-affected samples might result in a defective shortening process. Specifically, because the force for shortening is ATP-driven (Ertbjerg and Puolanne, 2017), with a reduced content of ATP present in the muscle, the sarcomeres are less likely to shorten because there is not enough energy to allow muscle contraction (Owens and Sams, 1997). Moreover, a study conducted by Mutryn et al. (2015) suggested that the higher concentration of reactive oxygen species detected in WB muscles may impact calcium release from the sarcoplasmic reticulum, damaging the ability of muscle cells to contract. Thus, it is reasonable to hypothesize that the hypoxic conditions along with the compromised energy-generating pathways of myopathic muscles might have consequences on the degree of muscle contraction, as *in vivo*, also during post-mortem. Moreover, considering these outcomes, it can be postulated that the typical hardness of WB muscles is not due to muscle fiber hypercontraction, otherwise, it is more likely linked to the excessive collagen deposition, as suggested in previous studies (Sihvo et al., 2014; Soglia et al., 2016; Baldi et al., 2019). Further, considering that sarcomere length is not uniform across the muscle (Moo et al., 2016), the spatial effect of WB myopathy on sarcomere length was investigated in different regions of PM muscle (anterodorsal, anteroventral, posteroventral, and posterodorsal) (Figure 11). Intriguingly, samples belonging to the anteroventral region of WB fillets showed the longest sarcomere length values (1.86 μm), while those belonging to the posterodorsal area exhibited the shortest (1.71 μm) and anteroventral and posteroventral displayed intermediate values (1.80 and 1.83 μm, respectively). Further, the superficial sections of both cranial and caudal regions (anteroventral and posteroventral, respectively) showed significantly longer (*P* < 0.05) sarcomeres when compared to their deep counterparts. These divergences detected along muscle’s thickness might be attributed to the different physical conformation and morphology of WB muscles, which usually show out-bulging and swollen areas in both cranial and caudal zones. In addition, considering fiber morphology results, anteroventral and posteroventral regions showed the most outstanding damages linked to WB disease (i.e., extreme collagen deposition, inflammatory cell infiltrations, endomysial, and perimysial spacing, etc.). Thus, it is reasonable to hypothesize that the profound injuries detected at histological level linked to a loss of muscle fiber functionality and vitality might have led to defective myofiber contraction mechanism *in vivo* that in turn resulted in longer sarcomeres.

## Conclusion

This study aimed to better understand the effects of WB condition on muscle post-mortem metabolism in an attempt to provide new insights about the factors contributing to the high pH_*u*_ of affected muscles. Reduced glycolytic potential only partially explains the higher pH_*u*_ in WB meat, as residual glycogen and PFK activity were unaffected in WB samples, arguing against glycogen content or PFK activity being the primary culprit for arresting glycolysis in WB. Further, the dramatic reduction in ATP concentrations in the early post-mortem period might suggest a defective ATP-generating pathway *in vivo.* It should be pointed out that the severe histopathologic damages associated to WB condition in general, and fiber necrosis in particular, might represent the logical culprits for the reduced ATP content found in WB. However, since ADP and AMP were not depleted by 24 h post-mortem, adenine nucleotides alone were not responsible for arresting post-mortem glycolysis. It has been also tested that the addition of excess ATPase through an *in vitro* glycolytic system can extend post-mortem glycolysis in WB muscles. In light of all the mechanisms involved in the occurrence of WB abnormality, it might be complex to define a single factor contributing to the higher pH_*u*_ of affected muscles. Considering the overall reduced glycolytic metabolites and the extreme myodegenerative processes associated with WB condition, data suggest that the higher pH_*u*_ of WB meat might be the outcome of a drastically impaired energy-generating pathway combined with a deficiency and/or a dysfunction of muscle ATPases, having consequences also on muscle fiber contraction degree.

## Data Availability Statement

The datasets generated for this study are available on request to the corresponding author.

## Ethics Statement

In this experiment, meat samples were directly collected post-mortem from carcasses belonging to animals that were farmed and slaughtered under commercial conditions in a federal slaughterhouse in Athens, Georgia (USA). Therefore, an ethical review process was not required for this study since the authors have not personally reared, killed nor handled the animals. All the aspects of farming, handling, transportation, and slaughter of birds were under the responsibility of the commercial slaughterhouse and accomplished under U.S. laws.

## Author Contributions

All authors listed have made a substantial, direct and intellectual contribution to the work, and approved it for publication. GB, DG, BB, HZ, and MP planned the experiment. GB designed the study, organized the databases and performed the statistical analysis, and wrote the first draft of the manuscript. GB, C-NY, MD, and JB performed the laboratory analyses and interpreted the results. All authors contributed to manuscript revision.

## Conflict of Interest

The authors declare that the research was conducted in the absence of any commercial or financial relationships that could be construed as a potential conflict of interest.
